# Acute Myocarditis Initially Presenting as Inflammatory Gastroenteritis With a Delayed Onset of Typical Chest Pain: A Diagnostic Challenge

**DOI:** 10.7759/cureus.104875

**Published:** 2026-03-08

**Authors:** Essam Elzoheiry, Mohammad Abdow, Abdalgany Al Ahmar

**Affiliations:** 1 Internal Medicine, Doha Clinic Hospital, Doha, QAT; 2 Internal Medicine, Turkish Hospital, Doha, QAT

**Keywords:** acute myocarditis, cardiac magnetic resonance, case report, gastrointestinal symptoms, inflammatory diarrhea, lake louise criteria, myopericarditis, troponin elevation

## Abstract

Acute myocarditis is an inflammatory disease of the myocardium with highly variable clinical presentations. While chest pain and dyspnea are common, atypical extracardiac manifestations may delay diagnosis, particularly in the absence of electrocardiographic abnormalities. Cardiac magnetic resonance imaging (CMR) has become the cornerstone for noninvasive diagnosis using the revised Lake Louise criteria.

A 37-year-old male presented with acute abdominal pain, inflammatory watery diarrhea, nausea, vomiting, and fever following recent food intake. Initial evaluation supported infectious gastroenteritis, including elevated fecal leukocytes and inflammatory markers. Shortly after admission, the patient developed chest discomfort, which prompted further cardiac investigation. Electrocardiography and echocardiography were normal. Serial high-sensitivity troponin-T levels subsequently demonstrated progressive elevation. CMR revealed myocardial edema and subepicardial late gadolinium enhancement involving the lateral and inferior walls, consistent with acute myocarditis. Typical inflammatory chest pain became more evident later in the clinical course. The patient was treated conservatively and discharged in stable condition.

This case highlights that myocarditis may initially mimic gastrointestinal disease. The emergence of chest discomfort or progressive troponin elevation should prompt further cardiac evaluation, even in the absence of early ECG abnormalities. Early use of CMR is essential for diagnosis in atypical presentations.

## Introduction

Acute myocarditis is an inflammatory condition of the myocardium with a broad clinical spectrum, ranging from subclinical disease to fulminant heart failure and sudden cardiac death [1]. The clinical presentation is heterogeneous, most commonly including chest pain, dyspnea, palpitations, or syncope, but atypical and extracardiac manifestations are increasingly recognized [2].

Myocarditis is most frequently caused by viral infections such as enteroviruses, adenoviruses, parvovirus B19, and SARS-CoV-2 (severe acute respiratory syndrome coronavirus 2) but may also result from autoimmune diseases, hypersensitivity reactions, or exposure to certain cardiotoxic drugs and toxins [2].

Cardiac troponins are sensitive biomarkers of myocardial injury and are frequently elevated in myocarditis, although their presence does not reliably correlate with the severity of ventricular dysfunction [3]. Importantly, troponin elevation may occur in the absence of ischemic symptoms or electrocardiographic changes, complicating the diagnostic process [2].

Cardiac magnetic resonance imaging (CMR) is the preferred noninvasive tool for diagnosing myocarditis, as it allows direct visualization of myocardial inflammation, swelling, and tissue injury [4]. The revised Lake Louise criteria, which combine T1 and T2 mapping markers, have markedly improved diagnostic accuracy, including in patients with preserved ventricular function [5,6].

Although gastrointestinal manifestations such as abdominal pain, vomiting, or diarrhea may accompany viral illness, gastrointestinal-dominant myocarditis is rare, reported in less than 10% of cases, and may lead to misdiagnosis as primary gastrointestinal pathology [7].

We report a case of acute myocarditis initially presenting as inflammatory gastroenteritis, with progressive troponin elevation preceding the onset of typical cardiac symptoms.

## Case presentation

A 37-year-old male presented to the emergency department with a two-day history of abdominal pain associated with frequent watery diarrhea, without blood or mucus. These symptoms were accompanied by nausea, a single episode of vomiting, and subjective fever. The patient reported recent consumption of restaurant food prior to symptom onset. He denied chest pain, dyspnea, palpitations, or localized chest discomfort at presentation.

His past medical history was significant only for gastroesophageal reflux disease. He had no known cardiovascular disease and was not taking regular medications.

On initial assessment, the patient was clinically stable. Vital signs showed a temperature of 36.7°C despite subjective fever, heart rate of 72 beats/min, blood pressure of 125/86 mmHg, and oxygen saturation of 99% on room air. Physical examination, including abdominal, cardiovascular, and respiratory systems, was unremarkable.

Given the patient’s stable clinical status, normal vital signs, and unremarkable physical examination, he was initially admitted with a provisional diagnosis of acute gastroenteritis. A stool analysis was obtained as part of the diagnostic workup and demonstrated the presence of leukocytes, suggesting an underlying inflammatory or infectious gastrointestinal process. Accordingly, the patient was managed conservatively as a case of presumed gastroenteritis and was started on supportive therapy.

The patient was initially managed as acute infectious gastroenteritis with intravenous normal saline, paracetamol 500 mg, and metronidazole 1 g.

However, following admission, the patient began to report new-onset chest discomfort, which prompted further evaluation. Additional laboratory investigations were therefore requested, including cardiac biomarkers, to assess for possible cardiac involvement.

Given the context of systemic inflammation and new-onset chest discomfort during observation, high-sensitivity troponin-T was specifically ordered to exclude occult myocardial injury or sepsis-related cardiac involvement.

Laboratory evaluation revealed markedly elevated inflammatory markers accompanied by significant elevation of cardiac biomarkers and D-dimer. Renal indices showed a mild rise in urea with preserved creatinine, indicating maintained renal function. Overall, these findings are consistent with an acute inflammatory state with biochemical evidence of myocardial injury in the absence of laboratory features suggestive of heart failure or coagulopathy (Table 1).

**Table 1:** Laboratory tests Biochemical and coagulation laboratory tests. CRP: C-reactive protein, Troponin-T HS: High-Sensitivity Troponin T, INR: International Normalized Ratio. The admission laboratory profile shows marked systemic inflammation (CRP, leukocytosis) with unexpectedly elevated high-sensitivity troponin-T and D-dimer.

Parameter	Result	Reference range
CRP	119.8 mg/L	0.0-5.0 mg/L
D-dimer	0.96 mg/L	0.00-0.49 mg/L
Troponin-T HS	178 ng/L	3-15 ng/L
Urea	8.2 mmol/L	2.5-7.8 mmol/L
Creatinine	94 µmol/L	62-106 µmol/L
INR	1.1	0.8-1.2

Blood cultures for both aerobic and anaerobic organisms were obtained to evaluate for potential systemic infection. Cultures were performed on admission and repeated during the course of hospitalization. By day five, all cultures remained sterile, showing no growth of pathogenic organisms, thereby ruling out bacteremia.

**Figure 1:**
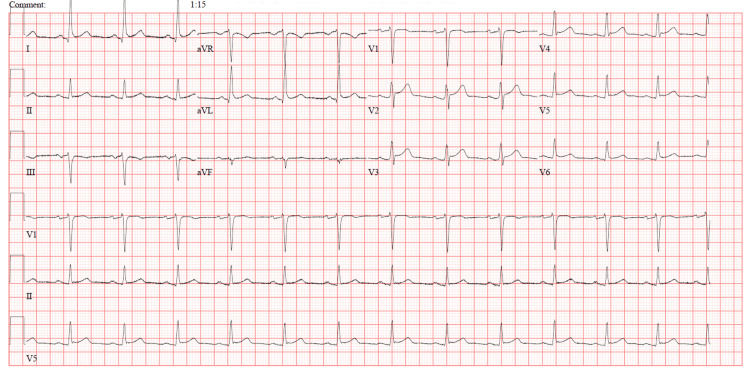
Electrocardiogram 12-lead electrocardiogram showing normal sinus rhythm with no ST-segment changes, T-wave inversions, or arrhythmias.

An admission electrocardiogram showed normal sinus rhythm with no ischemic or inflammatory changes (Figure 1).

Unexpectedly, high-sensitivity troponin-T was elevated with a progressive rise over subsequent days. Meanwhile, inflammatory markers gradually declined (Table 2).

**Table 2:** Troponin-T high-sensitivity trending High-Sensitivity Troponin T trending during patient hospitalization. Serial high-sensitivity troponin-T demonstrating progressive rise despite declining inflammatory markers, consistent with evolving myocardial injury. Troponin-T HS: high-sensitivity troponin T.

Parameter	Day one	Day two	Day three	Day four	Reference range
Troponin-T HS	178 ng/L	438 ng/L	1248 ng/L	1532 ng/L	3 – 15 ng/L

Transthoracic echocardiography demonstrated preserved left ventricular (LV) systolic function (auto-biplane LVEF 53%), normal diastolic function, LV global peak longitudinal strain of −18%, and no regional wall motion abnormalities or pericardial effusion.

The differential diagnosis at this stage included acute coronary syndrome, sepsis-induced myocardial injury, pulmonary embolism, and myocarditis. Acute coronary syndrome was deemed less likely given the absence of typical ischemic features, normal ECG, and preserved ventricular function.

**Figure 2:**
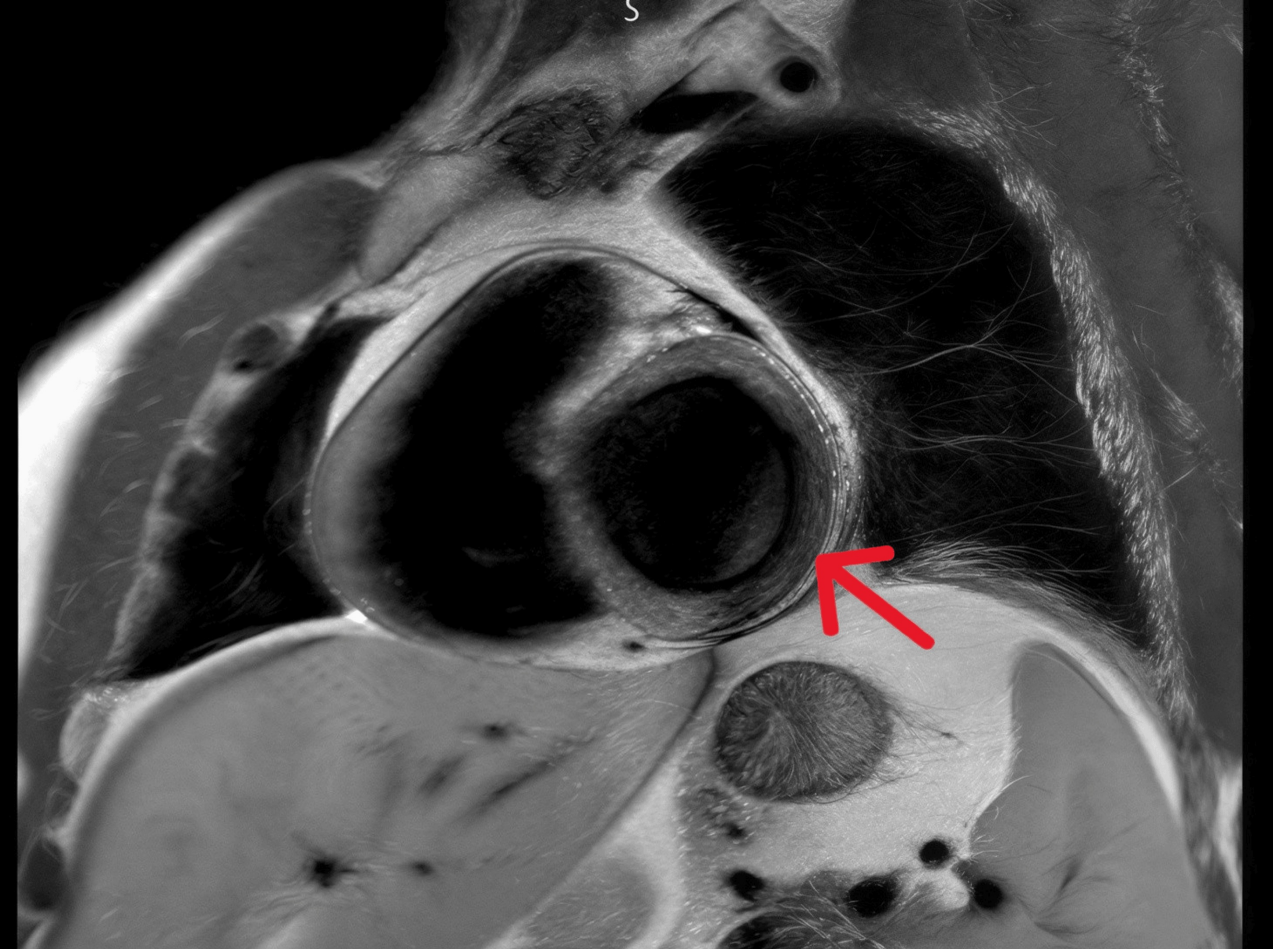
Cardiac magnetic resonance image The arrow indicates patchy myocardial edema with subepicardial early and late gadolinium enhancement involving the lateral and inferior left ventricular walls, a pattern that is typical of inflammatory myocardial injury, consistent with acute myocarditis.

Given the unexplained troponin elevation, CMR was performed two days after admission. It revealed normal biventricular volumes and systolic function (LVEF 55%, RVEF 55%), with patchy myocardial edema and subepicardial early and late gadolinium enhancement in the lateral and inferior walls, fulfilling the revised Lake Louise criteria for acute myocarditis (Figure 2) [1,6]. These findings confirmed nonischemic inflammatory myocardial injury, thereby obviating the need for immediate coronary angiography in this hemodynamically stable patient.

**Figure 3:**
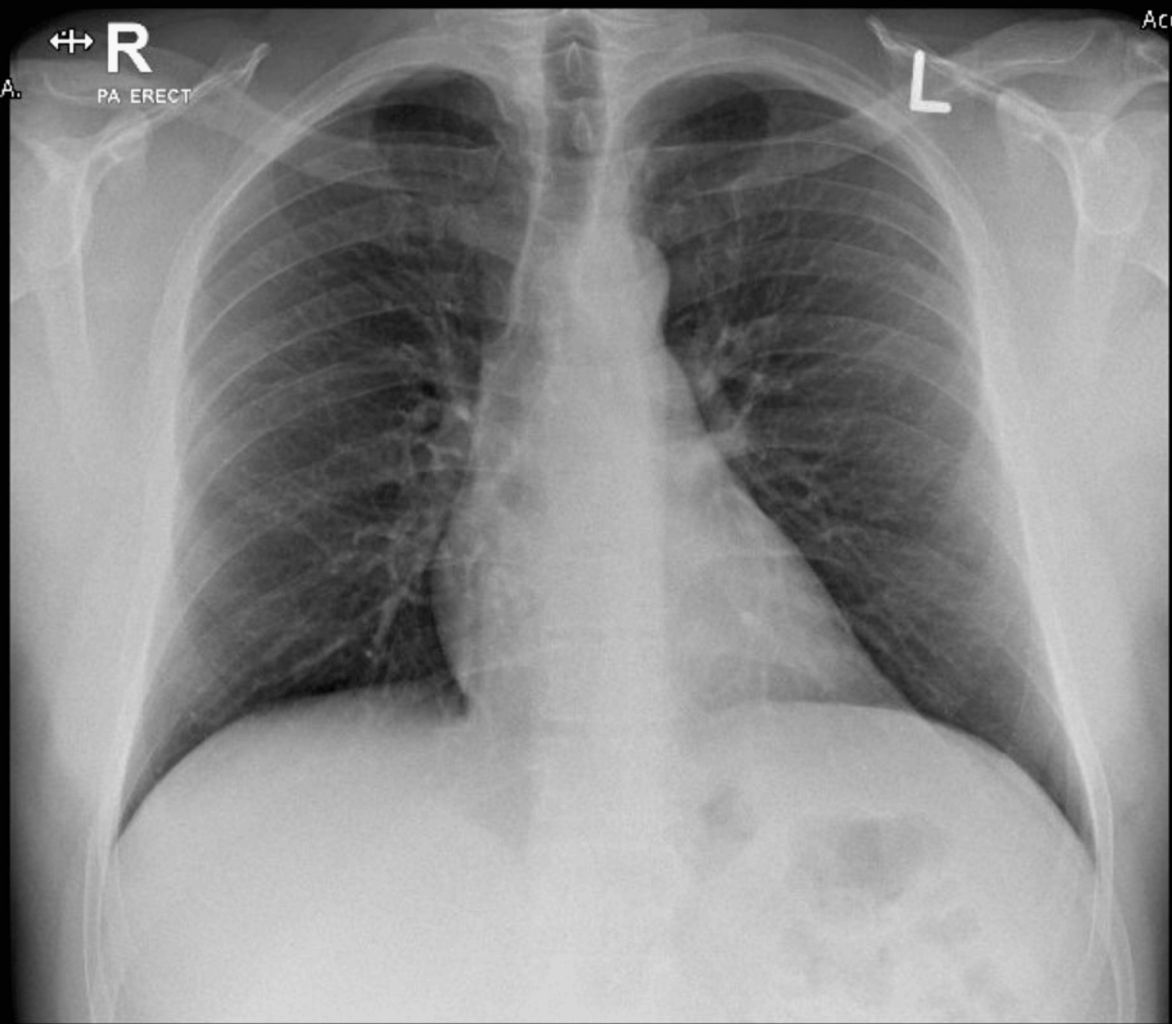
Chest X-ray Posterior-anterior chest radiograph demonstrating clear lung fields with no evidence of pulmonary congestion, consolidation, or pleural effusion.

Following diagnosis, the patient developed retrosternal pleuritic chest pain and nocturnal dyspnea, with pain relief on leaning forward, suggestive of myopericardial involvement. No new ECG changes or pericardial effusion were observed (Figure 3).

The patient was treated with colchicine and ibuprofen, remained hemodynamically stable, and experienced gradual symptom resolution. He was discharged on colchicine for three months and ibuprofen for one week, with outpatient cardiology follow-up.

## Discussion

Myocarditis is a challenging diagnosis due to its variable and often nonspecific clinical presentation [2].

This case specifically illustrates the need for cardiac evaluation in patients with systemic inflammation who develop chest discomfort and persistent troponin elevation during hospitalization for presumed gastroenteritis, rather than advocating routine cardiac biomarker testing in all gastroenteritis cases.

While chest pain is a common symptom, extracardiac manifestations may dominate the early clinical picture, leading to diagnostic delay [1].

In this case, myocarditis initially manifested as inflammatory gastroenteritis, supported by elevated fecal leukocytes and CRP. Gastrointestinal symptoms have been described in viral and systemic inflammatory illnesses associated with myocarditis but are rarely the dominant presenting feature [8].

The pivotal diagnostic clue was the progressive elevation of cardiac troponin. Troponin release reflects myocardial injury and is commonly elevated in myocarditis, even in the absence of ECG changes or ventricular dysfunction [3]. This underscores the importance of considering myocarditis in patients with unexplained troponin elevation and systemic inflammation.

Cardiac MRI was essential in confirming the diagnosis, demonstrating myocardial edema and subepicardial late gadolinium enhancement, a pattern typical for inflammatory myocardial injury and distinct from ischemic heart disease [4,5]. The revised Lake Louise criteria have improved sensitivity and specificity for myocarditis diagnosis, particularly in patients with preserved systolic function [6].

The delayed development of typical inflammatory chest pain in this patient suggests disease evolution along the myopericarditis spectrum, which may occur even in the absence of pericardial effusion or ECG changes [1]. Conservative anti-inflammatory therapy remains the cornerstone of management in stable patients [9].

However, this case has important limitations. Acute coronary syndrome was not formally excluded through coronary angiography or CT angiography. However, the clinical presentation (atypical chest discomfort, young age, low cardiovascular risk), normal ECG without ischemic changes, preserved biventricular systolic function, and CMR findings characteristic of nonischemic myocarditis strongly favored an inflammatory etiology. Additionally, no specific infectious etiology was confirmed despite negative blood cultures, with presumed viral gastroenteritis based on the clinical context.

## Conclusions

This case underscores the diagnostic challenge posed by acute myocarditis when it initially presents with isolated gastrointestinal manifestations and no early cardiac symptoms, electrocardiographic abnormalities, or ventricular dysfunction. The later onset of chest discomfort represented a pivotal diagnostic clue that shifted clinical suspicion toward myocardial involvement. Such evolution highlights that reliance on the initial presentation alone may delay recognition. Serial troponin assessment proved critical in raising suspicion, while CMR was decisive in establishing the diagnosis. Clinicians should maintain a high index of suspicion for myocarditis in patients with unexplained biomarker elevation or evolving cardiothoracic symptoms, even when the initial clinical picture suggests a noncardiac condition, as early identification directly influences monitoring, risk stratification, and outcomes.
